# *Leptospira* and Inflammation

**DOI:** 10.1155/2012/317950

**Published:** 2012-10-21

**Authors:** C. F. Gonçalves-de-Albuquerque, P. Burth, A. R. Silva, M. Younes-Ibrahim, H. C. Castro-Faria-Neto, M. V. Castro-Faria

**Affiliations:** ^1^Laboratório de Imunofarmacologia, Fundacão Oswaldo Cruz, FIOCRUZ, Rio de Janeiro 21040-900, Brazil; ^2^Departamento de Biologia Celular e Molecular, Instituto de Biologia, Universidade Federal Fluminense, Niterói 24020-150, Brazil; ^3^Departamento de Medicina Interna, Faculdade de Ciências Medicas, Universidade do Estado do Rio de Janeiro, Rio de Janeiro 20550-900, Brazil

## Abstract

Leptospirosis is an important zoonosis and has a worldwide impact on public health. This paper will discuss both the role of immunogenic and pathogenic molecules during leptospirosis infection and possible new targets for immunotherapy against leptospira components. *Leptospira*, possess a wide variety of mechanisms that allow them to evade the host immune system and cause infection. Many molecules contribute to the ability of *Leptospira* to adhere, invade, and colonize. The recent sequencing of the *Leptospira* genome has increased our knowledge about this pathogen. Although the virulence factors, molecular targets, mechanisms of inflammation, and signaling pathways triggered by leptospiral antigens have been studied, some questions are still unanswered. Toll-like receptors (TLRs) are the primary sensors of invading pathogens. TLRs recognize conserved microbial pattern molecules and activate signaling pathways that are pivotal to innate and adaptive immune responses. Recently, a new molecular target has emerged—the Na/K-ATPase—which may contribute to inflammatory and metabolic alteration in this syndrome. Na/K-ATPase is a target for specific fatty acids of host origin and for bacterial components such as the glycolipoprotein fraction (GLP) that may lead to inflammasome activation. We propose that in addition to TLRs, Na/K-ATPase may play a role in the innate response to leptospirosis infection.

## 1. Introduction

Leptospirosis is a zoonosis of global importance caused by several species and more than 200 different serovars of pathogenic* Leptospira spp*. The disease affects both animals and humans and has veterinary, economic, and medical relevance [1, 2]. Leptospirosis is still a major public health problem in tropical countries, with epidemic outbreaks occurring in the rainy season and after floods [3–5]. The annual incidence of this disease is estimated at 10–100 per 100,000 in tropical regions and 0.1–1.0 per 100,000 in temperate areas [6]. In recent years, leptospirosis outbreaks have occurred all over the world; thus, an adequate disease notification system would be useful to create surveillance networks [7]. Leptospirosis is transmitted to humans primarily by water contaminated with the urine of either wild or domestic mammals that have been chronically colonized by *Leptospira spp *[8]. It has recently been reported that *Leptospira* can persist in certain organs, indicating that people themselves can act as hosts [9]. 

In developed countries, the transmission mechanism is mainly associated with occupational and recreational activities [10–14]. The infection may be nonsymptomatic or may result in different clinical conditions ranging from a mild “flu-like” disease to a severe form known as Weil's disease [15–19]. Icterohemorrhagic syndrome is a severe form of leptospirosis in which symptoms comprise hepatitis, hemorrhage, acute lung injury, and renal failure [3, 18, 20, 21].

The leptospiral genome is greater than that of other spirochetes such as *Treponema sp*, which may explain the ability of *Leptospira* to live in several different environments and hosts [22, 23]. *Leptospira* species were recently grouped according to their genetic homology [24, 25], and studies aimed at the development of an efficacious vaccine are underway [26, 27].

After reaching the blood stream, spirochetes preferentially colonize the liver and kidney [28]. These organs can offer a large lipid supply because fatty acids are an essential requirement for leptospiral growth [29, 30]. There is evidence that leptospiras form a biofilm during kidney colonization in the proximal renal tubule lumen of *rabbit novergicus* [31]. Leptospiras can, however, also be found in other organs such as the lung and central nervous system [29, 30]. 

## 2. Pathogenesis 

Toxin production and/or the host immune response seem to be the main pathogenic mechanisms in leptospirosis. Like other spirochetes, leptospiras have a distinctive double membrane architecture that shares characteristics of both Gram-positive and Gram-negative bacteria [32].

A large proportion of the structural and functional outer membrane proteins (OMPs) is either lipoproteins such as LipL 32, LipL 21, and LipL 41 [33] or integral membrane proteins such as the porin OmpL1 [34]. In particular, OMPs may play key roles in pathogenesis by acting as adhesion or antigenic targets for bactericidal antibodies, receptors for various host molecules, and/or porins. Recent studies using five independent experimental methods have identified four novel surface-exposed and membrane-integrated leptospiral proteins (OmpL36, OmpL37, OmpL47, and OmpL54), although no functional roles have been described for them [35]. OmpA70 was identified in *L. interrogans* serovar Copenhageni [36] and the Lsa66 is a novel OmpA-like protein with dual activity that may promote the attachment of *Leptospira* to host tissues and may contribute to leptospiral invasion [37], indicating that OmpA-like proteins may have a role in leptospirosis pathogenesis.

 Virulence, characterized by mobility and the ability to invade tissues, may be associated with some lipopolysaccharides and adhesins [38–40]. Bacterial mobility likely plays a major role in the disease process of multiple spirochetes [41]. The ability to move rapidly in a sticky environment could contribute to the ability of the spirochete to cross through epithelial cells [38]. *In vitro,* pathogenic leptospiras penetrate the intercellular junction of endothelial cells while saprophytic *L. biflexa* do not [39]. The ability of leptospiras to penetrate and disseminate in mammalian tissue also depends on their ability to attach to cells and to the extracellular matrix. *In vitro*,* L. interrogans* binds to a variety of cell lines including fibroblasts, endothelial cells, and kidney epithelial cells [42]. 

Some proteins are potential virulence factors and have a role in bacterial adhesion to host tissues, such as the Lig protein and the leptospiral endostatin-like (Len) outer membrane proteins [43, 44]. Pathogenic leptospiras also express surface-exposed proteins that possess bacterial immunoglobulin-like domains such as LigA, LigB, and LigC, which are adhesin candidates [45]. Recent work has shown that LigB binds fibrinogen and inhibits fibrin formation [46]. Several groups have reported that immunization with the LigA-unique region conferred protection from lethal infection in both a mouse model [47] and a hamster model [48, 49] of leptospirosis. In addition, resistance in hamsters seems to depend on an immunity against a conformational epitope of Lig A that includes domains 11 and 12 and a third flanking domain (either 10 or 13) that may be required for proper conformational folding [50]. Moreover, the endostatin-like protein A (Len A) was shown to bind to the host component laminin [51] and to human plasminogen [52].

Comparative studies of different serovar genomes have suggested that other components such as integrin alpha-like protein (also an adhesin candidate), lipopolysaccharides, cell surface capsular polysaccharides, and exopolysaccharides may also play a role in bacterial survival in specific host organs [22]. The OmpA-like protein Loa22 was reported to be essential for leptospiral virulence [53] and to promote inflammatory responses in cultured rat renal cells [54]. The virulence factor Loa22 is a highly conserved lipoprotein with a peptidoglycan-binding motif similar to OmpA that is upregulated during acute *leptospira* infection [19]. Hemoxygenase, FliY (flagellar motor switch protein), and LPS are other recognized virulence factors [32].

Other molecules that could play a part in leptospira infection include potential toxins such as the hemolysin SphH, a pore-forming protein without sphingomyelinase or phospholipase activities [55], and the enzyme catalase (KatE), which is produced only by pathogenic strains and is involved in resistance to oxidative killing [22, 56]. 

## 3. *Leptospira* Metabolism and Endotoxins

Leptospiras are strictly aerobic spirochetes. In their culture medium, they require ammonia as the nitrogen source [57] and long chain fatty acids as the sole carbon and fuel sources [58], and they obtain energy through the fatty acid *β*-oxidation pathway [29]. The most commonly used culture medium is Ellinghausen-McCullough/Johnson-Harris medium, which contains oleic acid, bovine serum-albumin, and polysorbate [19].

The biological activity of the lipopolysaccharide-like substance (LLS) extracted from the *L. interrogans* serovar canicola was weaker than the lipopolysaccharide (LPS) obtained from other gram-negative bacteria [59]. Lipid A is the active component of LPS and is responsible for its toxic activity. The lipid A of leptospiral LPS has an unusual fatty acid composition and, more strikingly, a unique methylated phosphate residue [60]. Leptospiral lipid A is structurally and functionally different than the lipid A of* E. coli* [61]. The glycolipoprotein fraction (GLP) is another leptospiral component that has cytotoxic activity [62]. 

Due to their peculiar metabolism, leptospiras are able to store lipids such as fatty acids [62, 63]. Some lipids are stored associated with GLP (palmitovacenic, linoleic, and oleic acids) [62], while others are stored associated with LPS and LLS (hydroxylauric, palmitic, and oleic acids) [64, 65]. These reports indicate that leptospiras are able to store and associate fatty acids with their endotoxins (LPS and GLP). This ability may have important pathophysiological consequences. 

## 4. Toll-Like Receptors and Immune Response in Leptospirosis

The innate immune response is based on the recognition of pathogen-associated molecular patterns (PAMPs) [66, 67]. Immune cells express proteins called pathogen recognition receptors (PRRs) that allow them to recognize conserved microbial motifs such as peptidoglycans and LPS [68–70]. 

TLR4 was the first PRR to be described and was identified in 1997 [71]. TLR4 shows a highly orchestrated usage of coreceptors to discriminate between ligands. This receptor signals the presence of LPS in association with the CD14 [72] and MD-2 proteins [73]. This multifaceted receptor system additionally plays a role in triggering several signal transduction pathways [74]. For example, LPS binding to TLR4 activates transcription factors such as the nuclear factor NF-*κ*B, which induces the production of inflammatory interleukins (IL-1*β*, IL-6, IL-8) and tumor necrosis factor (TNF) [69]. 

Another TLR, TLR2, is essential for the recognition of Gram-positive bacterium components such as the macrophage-activating lipopeptide 2 (MALP-2) and lipoarabinomannan, the main glycolipid of *Mycobacterium tuberculosis* [75]. In association with another TLR (TLR6), TLR2 triggers intracellular signaling through the mitogen-activated protein kinases (MAPKs) and NF-*κ*B [70]. 

During leptospirosis, bacterial recognition by host is under disclosure, but *Leptospira* presence may be sensed through TLR4 and TLR2 receptors [76]. 

It is well known that LPS from Gram-negative bacteria activates the TLR4 signaling cascade. Paradoxically, *L. interrogans *LPS binds both CD14 and TLR2 but does not generate intracellular signaling through TLR4 activation [77]. The lipid A from *Leptospira* LPS apparently stimulates mouse cells through the TLR4-MD2 complex but does not induce signaling in human cells [61], indicating that there are species-specific aspects of LPS signaling that differ between mouse and human cells.

 In recent years, considerable research has been conducted on the outer membrane proteins expressed by *Leptospira spp*. during infection. LipL32 is the major leptospiral outer membrane lipoprotein expressed during infection and is the immune-dominant antigen recognized in humoral responses against leptospirosis in humans [78, 79]. This lipoprotein is highly conserved among pathogenic *Leptospira *species [79] and signals through TLR2 [77], as recently confirmed by data showing the LipL32 binding to TLR2 in renal cells [80]. However, LipL32 was not required either for the development of acute leptospirosis in hamsters or for renal colonization in a rat model [81]. LipL21, the second major outer membrane protein of the *Leptospira interrogans* serovar *Lai*, exhibits potent immunogenic activity [82]. 

It has been reported that the *Leptospira santarosai* serovar Shermani activates the production of proinflammatory chemokines induced by p38 MAPK phosphorylation through TLR2 activation in proximal tubule epithelial cells in mice [83]. These same investigators also observed that OMPs and LipL32 increased TLR2 expression in human embryonic kidney cells (HEK 293). In addition, LipL32 augmented iNOS and CCL2/MCP1 mRNA expression and protein secretion via TLR2 binding [84]. 

The infection of guinea pigs with the *L. interrogans* serovar Icterohemorrhagiae increased the levels of IL-6 and TNF*α* mRNA in the lungs [85], and uveitis of leptospiral origin was associated with an increased production of the cytokines IL-6 and IL-8 [86]. An increase in cytokine production was also linked to a lethal outcome in leptospirosis patients [87].

C3H/HeJ mice have deficient LPS signaling and only respond to high doses of LPS [88]. Animals unable to detect LPS appropriately are susceptible to infection by Gram-negative bacteria [66]. When C3H/HeJ mice were infected with the *Leptospira interrogans* serovar icterohemorrhagiae, they presented with a lethal infection with morphological changes in the kidney and lungs [89] as well as sustained expression of CCL2/MCP-1 and CXCL1/KC in the lungs, which were correlated to the severity and progression of disease [90]. Another strain of mice, C57BL/10ScCr, carries a null TLR4 mutation, does not express TLR4 protein, and is resistant to high doses of LPS [88]. These animals do not express the receptor to IL-12p40. Both C3H/HeJ and C3H/SCID mice presented with a lethal outcome when infected with the *Leptospira interrogans* serovar Copenhageni [91]. The C3H/HeJ animals died after an intraperitoneal injection of *Leptospira interrogans* serovar *icterohemorrhagiae, *presenting with liver disease and lung hemorrhage [92].

Virulent leptospiras can protect themselves against components of the host's innate immune system, such as phagocytic cells and the complement system. Pathogenic leptospiras escape from phagocytosis and are resistant to intracellular killing mechanisms [93, 94]. To establish a successful leptospirosis infection, the leptospiras must be able to evade the complement system. In contrast, nonpathogenic leptospiras are killed after exposure to the human complement system [95]. It has been shown that the acquisition of factor H (FH) and other complement modulators displayed on the *Leptospira* surface is crucial for bacterial survival in serum. Leptospiras isolated from patients can bind the complement system inhibitor FH, a regulatory complement protein that prevents complement activation, and can restrict the deposition of the late complement components on their surfaces [96]. Thus, binding of this major alternative complement pathway inhibitor is related to serum resistance in *Leptospira* spirochetes. Interestingly, FH binding was shown to be dependent upon Lig proteins [97]. The multifunctional LigB protein also binds to C3b and C4b and interferes with complement activation [98]. Lsa30, a novel leptospiral adhesion protein, may help pathogenic *Leptospira* to escape the immune system by interfering with the complement cascade through interaction with the C4bp regulator [99]. Lsa33 also bind to C4bp and may be important in immune evasion [100]. The recently described LcpA (leptospiral complement regulator-acquiring protein A) also binds to C4bp [101]. 

Acquired immunity that is protective against reinfection by *Leptospira* does occur, but this has been shown in animal models to be dependent on the specific *Leptospira* serovar [102]. Specific antibodies to *Leptospira* membrane proteins may play a role in host defense [103] in animal vaccination models. Vaccines prepared with the LipL21 antigen protected guinea pigs from leptospiral infection [82], but there is currently no consensus regarding which signaling pathway is involved. Recent work showed that murine B cells were crucial to clearing *Leptospira*, through both early IgM production against LPS, which depends on TLR4, and protective IFN*γ* production, which depends on TLR2 and TLR4 activation [104]. It has also been shown that cattle immunized with a killed *Leptospira* vaccine develop protective immunity associated with CD4+ T cells and *γδ*T cells [105]. Nevertheless, patients who have recovered from leptospirosis do not seem to generate memory T cells that can be activated by *in vitro *stimulation with Leptospiral protein antigens [106]. 

## 5. New Insights

**Figure 1:**
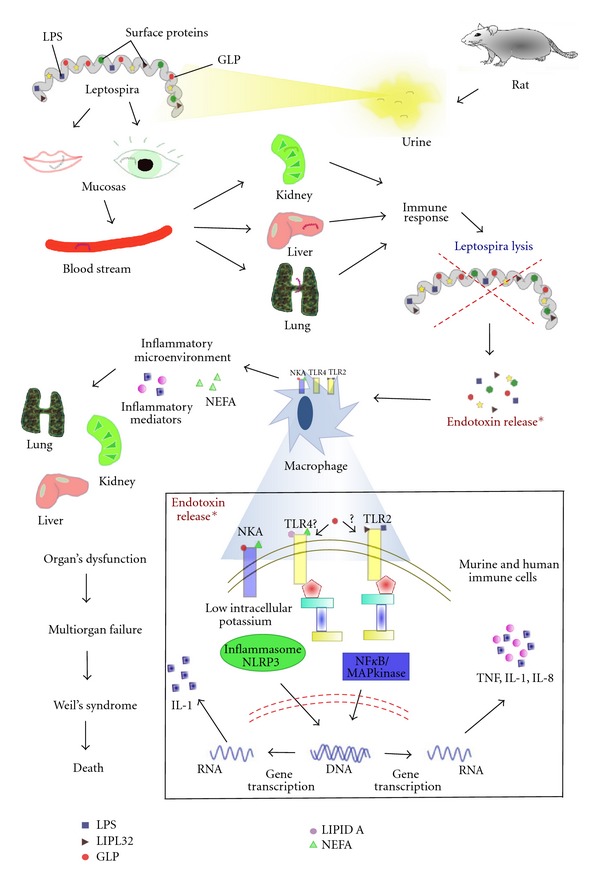
Severe leptospirosis: from the infection to immunological target. Due to their mobility, leptospiras are able to penetrate mucosal tissues and injured skin. Transported by the blood stream, they reach target organs, mainly the kidney and liver. The host immune response kills the bacteria, promoting endotoxin release. The innate immune system of both human and mouse recognizes endotoxins through specific receptors. This immune cell response is mediated by Toll-like receptors and Na/K-ATPase, which sense antigen molecules and trigger intracellular signaling pathways driving the translocation of transcription factors, leading to increased inflammatory mediator production. This scenario creates an inflammatory microenvironment that can lead to organ dysfunction. Another important observation in this disease is the increased NEFA levels in the systemic circulation (mainly oleic acid). Augmented albumin unbound-NEFA may play an important role in multiorgan dysfunction by acting on endothelium and immune cells. TLR2: Toll-like receptor 2; TLR4: Toll-like receptor 4; NKA: Na/K-ATPase; NF-*κ*B: nuclear factor kappa-light-chain-enhancer of activated B cells; NEFA: nonesterified fatty acid; LIPL32: major outer membrane leptospiral lipoprotein; GLP: leptospiral glycolipoprotein.

When humans come in contact with contaminated water or soil, pathogenic leptospirasenter the blood stream either via skin lesions or by actively penetrating the mucosa and colonizing organs such as the kidney and liver (Figure 1). Meanwhile, the immune system induces bacterial lysis, releasing many antigens, including the glycolipoprotein GLP and LPS.

The hypothesis that *Leptospira* produces an endotoxin released after bacterial lysis due to the host immune response was investigated and is supported by clinical and histopathological observations [107]. Nevertheless, the severity of Weil's syndrome seems to be related not only to the virulence and toxin liberation from the infective serovar but also to the intensity and the speed of the host immune response [3, 108]. The production of specific antibodies is essential to protect mice from *Leptospira* infection because macrophages can only efficiently phagocytose leptospiras in the presence of a specific antibody [109]. The *L. interrogans* GLP is also released by bacterial lysis and can activate inflammatory cells, such as peripheral blood mononuclear cells (PBMC), leading to an increased production of TNF*α* and IL-6 [16], an increased expression of the adhesion molecule CD69, and an augmented secretion of prostaglandin E_2_, leukotriene B_4_, and nitric oxide [110].

Acute lung injury (ALI) is characterized by cytokine release and the loss of epithelium/endothelium integrity. The increased permeability leads to protein extravasation and edema. This is the hallmark of all ALI/ARDS [111]. The presence of leptospiras and leptospiral antigens in lung endothelial cells is thought to be evidence that pulmonary lesions are triggered by bacteria and their toxic products [3, 112, 113]. Patients with fatal leptospirosis generally suffer extensive pulmonary hemorrhage [114]. *Leptospira* infections in monkeys mimic the features of severe human leptospirosis, including pulmonary hemorrhage [115]. The pulmonary hemorrhage is thought to be linked to the deposition of immunoglobulin and complement in the alveolar septa [116]. Pulmonary hemorrhage is a serious life-threatening disorder and is the major cause of death due to leptospirosis in Brazil [18].

In the lung, the enzyme adenosine triphosphatase is activated by Na^+^, K^+^, and Mg^++^ (Na/K-ATPase) and removes sodium from alveolar fluid, contributing to edema clearance and acting as a homeostatic mechanism to maintain lung integrity [117–119]. Inhibition of the Na/K pump in this organ may contribute significantly to lung failure in severe cases [120]. The kidney is another important leptospiral target, and acute kidney injury is an early manifestation of leptospirosis [121]. Inhibition of the Na/K pump in the kidney leads to loss of potassium and to hypokalemia [122]. Indeed, acute renal failure in leptospirosis is initially characterized by hypokalemia [123, 124]. Dysfunctional Na^+^ transporters in the kidney and lung have already been observed in the context of this disease [125]. Interestingly, engulfed GLP has been detected in phagocytes in the kidney [126] and, as we have demonstrated, is a specific Na/K-ATPase inhibitor [127]. 

The liver is another organ that is affected in leptospirosis infections. Inhibition of Na/K-ATPase in liver contributes to liver functional disorder and causes decreased albumin and increased nonesterified fatty acids (NEFA) and bilirubin in the plasma [127]. We also showed that this inhibition may be caused by nonesterified monounsaturated fatty acids (NEUFA) such as oleic and linoleic acids, which are GLP components and are substantially augmented in the plasma of patients with severe leptospirosis [128]. High NEFA levels are characteristic of patients with severe leptospirosis and other inflammatory conditions [128]. Increased circulating levels of NEFA also occur in some respiratory diseases, and as NEFA are known to be immune-stimulatory agents [129], this increase may directly contribute to systemic inflammation and more severe disease by stimulating the production of inflammatory mediators [130]. High levels of circulating NEFA can either inhibit or activate TLR4, triggering the inflammatory response [131]. Similar to LPS, saturated fatty acids can induce inflammatory responses in dendritic cells [132], although polyunsaturated fatty acids negatively modulate TLR4 [133]. Fatty acids such as lauric, palmitic, and oleic acids activate TLR4 in adipocytes and macrophages, leading to augmented IL-6 and TNF*α* production [130]. Furthermore, NEFA binding to free fatty acid receptors stimulates intracellular responses, augmenting the formation of inflammatory mediators [134, 135] via the activation of NF-*κ*B and AP-1, as demonstrated in human endothelial cells [136]. 

Recently, Na/K-ATPase has been described as a receptor for intracellular signaling cascades. In this novel role, the enzyme functions as a receptor for nanomolar ouabain concentrations and other cardiac glycosides and triggers intracellular signaling cascades without changing the intracellular Na^+^ and K^+^ concentrations [137, 138]. Protein interactions with Na/K-ATPase have an important role in membrane rafts, which are linked to calcium signaling [139], and can be released through IP3 receptor binding [140]. In the presence of ouabain, calcium oscillations lead to NF-*κ*B activation [141] and ERK/MAPK activation, which may lead to the activation of the transcription factor AP-1 [142]. The ouabain effects in signal transduction occur through a pool of Na/K-ATPase without interfering with pump activity [143]. In this respect, it was demonstrated that ouabain acts on lymphocytes without depolarizing the membrane, suggesting a mechanism that is independent of classic pump inhibition [144]. 

Na/K-ATPase binding triggers intracellular pathways that lead to the production of proinflammatory mediators [136, 137]. The binding of ouabain to Na/K-ATPase induces mononuclear cells to secrete TNF-*α* and IL-1 [145]. In the context of inflammatory leptospirosis, monocytes stimulated by leptospiras and their extracts respond by activating intracellular pathways, phosphorylating p38, activating NF-*κ*B, and releasing cytokines and nitric oxide [94, 146]. The relevance of inflammatory mediators to the physiopathology of experimental and clinical leptospirosis is well known. Hamsters infected with *L. interrogans sorovar Icterohemorrhagiae *that exhibit lung injury had increased mRNA levels of TNF and IL-6 [85]. Components of *Leptospira* are able to induce TNF release [147]. The *L. interrogans *GLP, a bacterial fraction that inhibits Na/K-ATPase [122, 127, 148], is able to induce inflammatory cell activation and increase TNF*α* and IL-6 production [16]. Increased TNF production is a predictor of poor clinical outcome in patients with leptospirosis [149]. Furthermore, the uveitis seen in leptospirosis is associated with a rise in IL-6, IL-8, TNF-*α*, and IL-10 production [86]. Increased cytokine production is associated with increased patient mortality during the disease progression [87]. IL-1*β* and IL-18 are produced by inflammasome activation [150]. The inflammasome consists of several proteins, of which NLRP3 is involved in the recognition of bacterial RNA, ATP, uric acid, and low intracellular potassium concentrations [151]. A recent report showed that *Leptospira* induces production of the cytokine IL1*β* through synergy between LPS signaling via TLRs and leptospiral GLP, which inhibits the Na/K ATPase, triggers a decrease in intracellular potassium levels, and activates the NLRP3 inflammasome [152]. Thus, it is possible that the increased production of inflammatory mediators in leptospirosis is related both to recognition mechanisms involving TLR4 and fatty acid receptors and to a mechanism dependent on Na/K-ATPase signaling. In this way, both GLP and ouabain inhibit Na/K-ATPase and induce the production of inflammatory mediators directly involved in the pathophysiology of leptospirosis.

 We cannot dismiss the hypothesis that GLP, also a specific Na/K-ATPase inhibitor, and the increased NEFA concentrations observed in the plasma of leptospirosis patients, represent a novel mechanism of triggering the inflammatory cascade, leading to the exacerbation of the immune response associated with the multiorgan dysfunction observed in this disease.

## 6. Final Remarks

 In summary, the existing data still form an incomplete picture. TLR4 seems to be a crucial effector in the fight against *Leptospira* and is directly involved in the development of resistance to leptospiral infection. TLR2 also has an important role in leptospiral protein and LPS recognition. Furthermore, both TLR4 and TLR2 seem to be involved in the protection against pathogenic *Leptospira *antigens. Although TLR4 and TLR2 are directly implicated in the immune response to this disease, other mechanisms could be involved in the recognition of leptospiral molecular patterns. Some candidates are now emerging.

*Leptospira* components that are directly released after bacterial lysis may be involved in the pathophysiology of this disease either by causing direct injury or by triggering inflammation. In this respect, Na/K-ATPase alterations caused by GLP binding or by increased plasma levels of NEFA can trigger direct or indirect damage through the exacerbation of the inflammatory response. 

## Abbreviations

TLR: Toll-like receptor
LipL: Leptospiral outer membrane lipoprotein
OMP: Outer membrane protein
GLP: Glycolipoprotein fraction
FliY: Flagellar motor switch protein
KAtE: Enzyme catalase
PRR: Pathogen recognition receptor
(LLS): Lipopolysaccharide-like substance
(LPS): Lipopolysaccharide
IL: Interleukin
TNF: Tumor necrosis factor
MALP-2: Macrophage-activating lipopeptide 2
MAPK: Mitogen-activated protein kinase
HEK 293: Embryonic kidney cells
(PBMC): Peripheral blood mononuclear cells
ALI: Acute lung injury
NEFA: Nonesterified fatty acids
NEUFA: Nonesterified monounsaturated fatty acids
AP-1: Activator protein
NF-*κ*B: Nuclear factor kappa-light-chain-enhancer of activated B cells.
